# Features of the Microstructure and Chemical Compositions of Vanadium-Containing Slags Including Determination of Vanadium Oxidation Degrees

**DOI:** 10.3390/ma12213578

**Published:** 2019-10-31

**Authors:** Anatoly Kovalev, Dmitry Wainstein, Vladimir Vakhrushev, Anton Volkov, Ulyana Kologrieva

**Affiliations:** State Scientific Centre, I.P. Bardin Central Research Institute for Ferrous Metallurgy, 23/9 bdg. 2, Radio str., 105005 Moscow, Russia; a_kovalev@sprg.ru (A.K.); gareq1211@gmail.com (V.V.); rhenium@list.ru (A.V.); ufowka@mail.ru (U.K.)

**Keywords:** vanadium, converter slag, extraction, oxidation degree, phase composition, X-ray fluorescence analysis (XRF), scanning electron microscopy (SEM), X-ray photoelectron spectroscopy (XPS)

## Abstract

Metallurgical vanadium-containing converter slag could be used as an alternative vanadium source. The development of a physico-chemical basis for the comprehensive processing of industrial vanadium-containing debris requires information about their elemental composition as well as the oxidation degrees of the elements and forms of compounds in order to solve two key problems: a better utilization of industrial wastes and a lowering of environment impact. This research was aimed at the development of methods to determine the fractions of elements and their oxidation degrees in vanadium-containing industrial debris exemplified by basic oxygen converter vanadium slags. A set of bulk and surface analysis methods (X-ray fluorescence analysis (XRF), scanning electron microscopy (SEM), X-ray photoelectron spectroscopy (XPS)) was used for this purpose: based on results of elemental analysis, SEM detects the oxide phases of metals, while an analysis of the XPS lines’ fine structures provides fractions of corresponding elements with definite oxidation degrees. In this way, one can determine the fractions of vanadium in multiple oxidation degrees in slags and can properly select the chemicals and parameters of chemical processes for its fullest extraction.

## 1. Introduction

Vanadium is considered to be one of the prospective alloying metals that can produce steels with high mechanical and other consumer properties. Approximately 85% of the consumed vanadium relates to ferrovanadium [1]. Most vanadium is extracted from titanium magnetite ores. Currently, ferrovanadium production technology includes four process stages: the fabrication of vanadium iron, the processing of the iron to a steel semi-product and a converter slag, the fabrication of technical vanadium pentoxide, and the electrical furnace or ladle melting of ferrovanadium [2,3,4,5,6,7,8,9]. There are also other options for vanadium extraction [10,11,12,13,14]. 

Vanadium slag is a strongly consolidated mass with metallic inclusions. An oxide part of the slag contains compounds of iron, silicon, manganese, vanadium, chromium, titanium, magnesium, aluminum, and calcium. Metal inclusions are formed by an iron-based alloy, and their dimensions can vary from fractions of millimeter to several tens of centimeters in diameter. Vanadium spinel is the main mineral in the slag. Its silicate part is comprised of fayalite, cristobalite, olivine, pyroxene, and glass. It is generally believed that the vanadium in the slag has a +3 oxidation degree, mainly in spinel [15].

Metallurgical slags, vanadium processing slams, vanadium-containing quartzites, shales, pitch [16], uranium ore [17], bauxites [18], ash residuals from power units burning heavy oil fractions [19], dead catalysts [20], the ash of coal power plants, etc., could be alternative vanadium sources. A hydrometallurgical fabrication of technical vanadium pentoxide is characterized by a high amount of wastes containing V compounds; the main ones are dump slimes resulting from the leaching of vanadium from calcinated burden [21]. According to [22], one ton of produced V_2_O_5_ generates about 200 tons of tails. Soluble V and Cr compounds pose a serious threat of environment pollution, especially when the latter is presented in the +6 oxidation degree.

The development of the physico-chemical basis for the comprehensive processing of industrial vanadium-containing debris requires information about their elemental composition as well as the oxidation degrees of elements and the forms of compounds in order to solve two key problems: a better utilization of industrial wastes and a lowering of environment impact. The present work was aimed at the development of methods to determine the fractions of elements and their oxidation degrees in vanadium-containing industrial debris exemplified by basic oxygen converter vanadium slags.

## 2. Materials and Methods 

The vanadium containing converter slags from Joint Stock Company Nizhniy Tagil Iron and Steel Works obtained from the smelts of different steel grades alloyed by V, Ti, and Mn were studied in this research. Five slag compositions with vanadium contents of more than 10 wt.% were selected for detailed investigations.

The elemental composition of the slags was determined using an AXIOS^max^ Advanced (PANalytical, Almelo, The Netherlands) X-ray fluorescence spectrometer. Probes were dispersed to diameter about 40 μm by an RS200 (Retsch, Haan, Germany) vibrational planetary mill; they were dried after milling and melted with a melting blend of lithium tetraborate, carbonate, and nitrate in a platinum crucible using an Eagon 2 (PANalytical, Almelo, The Netherlands) furnace. The elemental composition of the samples is presented in Table 1.

The phase composition of the slags in the initial state was determined by scanning electron microscopy. Metallographic specimens were prepared according standard ISO 4967:2013 procedure [23] on slag pieces with dimensions of about 20 mm × 20 mm × 20 mm. The specimens were cleaned after polishing by chemically pure acetone to avoid occasional surface contaminations. 

The sample surfaces were examined using a scanning electron microscope (SEM) JSM-35C (JEOL, Tokyo, Japan) at magnifications from ×30 to ×1000, and the studied areas on the samples’ surface were from several square millimeters to several microns, respectively. Five metallographic samples were investigated for each slag composition. 

Preliminary quantitative phase composition was investigated by scanning electron microscopy (SEM) with using Back Scattered Electrons image analysis (BEI) on JSM-35C with the solid pair detector attached. This method has been widely used in mineralogy from 1984 to the present [24]. The phase composition analysis was based on the distinctive contrast of each phases: The intensity of the backscattered electrons is directly proportional to the average atomic number (AAN) of the observed phases and is used for imaging distinguishing individual mineral grains and identifying the zones of discrete phases. For example, the AAN of Al_2_O_3_ = (2 × 13 + 3 × 8)/5 = 10; Fe_3_C will be darker than α-Fe.

Contrast resolution can distinguish differences as low as 0.1 AAN. High quality atomic number contrast images are produced providing much more useful information than those normally obtained from SEM. Compositional information dominates topographic information in the images, allowing for the differentiation of most phases. Metallurgical slag contains a set of known mineral compositions. Quantitative analysis is one problem in the investigation of a huge number of metallographic samples. BEI analysis is the most effective and simple analysis method in this case because wavelength dispersive (WD) and energy dispersive (ED) X-ray investigations are far too slow to be used for routine and representative mineral identification [25]. The microstructure image processing was done by ImageJ [26] software to obtain brightness distribution diagrams with typical peaks. The peak squares relations correspond to the volume fractions of phases in the slag sample. The gray scale segmentation of BEI was based on the principles described in [27].

The analysis of the oxidation degrees of elements in slags was performed by X-ray photoelectron spectroscopy (XPS) on ESCALAB Mk2 (VG, East Grinstead, UK) at a vacuum of 3 × 10^−8^ Pa. An electron spectrometer was equipped with an X-ray Al K_α_ monochromatic source (hν = 1486.6 eV, Au 4f3/2–5/2 full width at half maximum (FWHM) = 0.6 eV). Powder samples were used to get the averaged compositions. The powder was placed on the special sample holder using conducting carbon ribbon. The sample charge was suppressed by slow energy electron bombardment with an energy of 30 eV. The fine structure of XPS lines was analyzed using UNIFIT2007 [28] software. 

## 3. Results and Discussion

The phase composition analysis was based on typical contrast for each of phases. Brightness distribution histograms were obtained from micro images; the peak squares correspond to phases contents in the slag samples. Thus, the main peak on the histograms was associated with a matrix supposedly consisting of magnetite (FeO·Fe_2_O_3_). Relatively big iron particles from 50 to 350 μm were found on several microstructures. They had a light-grey contrast. On the SEM images, one can also see the vanadium-containing spinel MnO·V_2_O_3_ (bright contrast) and pores with polyhedral inclusions of TiO_2_ (Figures 2b, 3b, 4b, and 5a) single crystals with a characteristic faceting of the {001}, {101}-type and quasi-continuous microfacets [29]. The contrast on the SEM images is stipulated by so-called “atomic number contrast” arising from different reflection of secondary electrons from regions containing heavy and light elements with bigger and smaller AANs.

Thus, the AANs of the FeO·Fe_2_O_3_, MnO·V_2_O_3_, TiO_2_, and Fe phases presented in metallurgical slag are very different and equal to 15.7, 13.2, 12.7, and 26, respectively, that allowed us to easily identify them on the BEI images.

**Figure 1:**
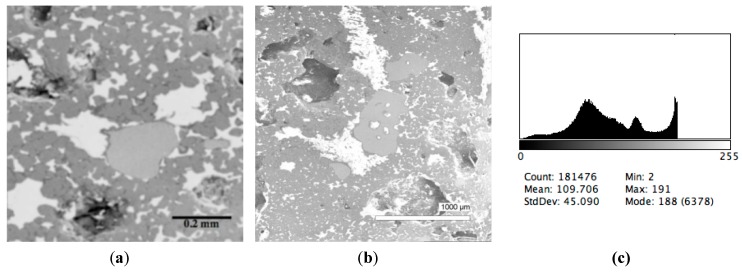
Typical back scattered electron images (**a**,**b**) of microstructures and contrast histogram (**c**) of the Sample #1 grind; scanning electron microscopy (SEM).

**Figure 2:**
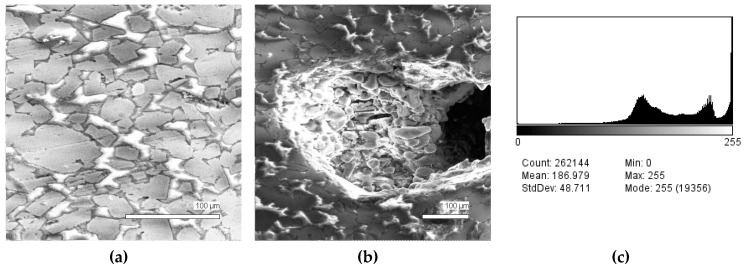
Typical back scattered electron images (**a**,**b**) of microstructures and contrast histogram (**c**) of the Sample #2 grind; SEM.

**Figure 3:**
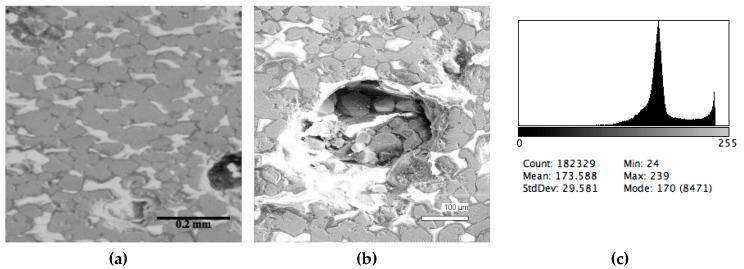
Typical back scattered electron images (**a**,**b**) of microstructures and contrast histogram (**c**) of the Sample #3 grind; SEM.

**Figure 4:**
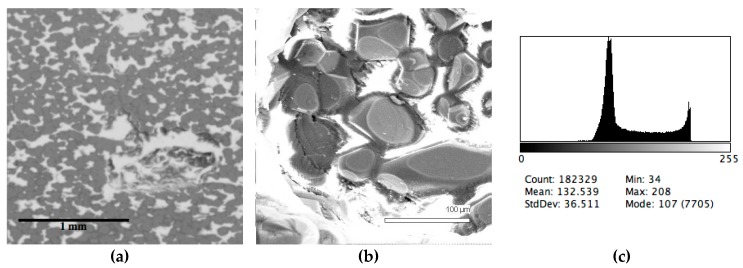
Typical back scattered electron images (**a**,**b**) of microstructures and contrast histogram (**c**) of the Sample #4 grind; SEM.

**Figure 5:**
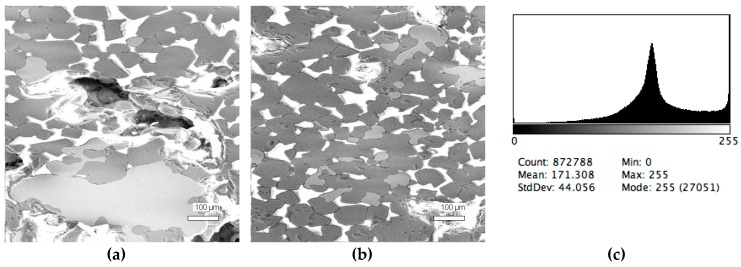
Typical back scattered electron images (**a**,**b**) of microstructures and contrast histogram (**c**) of the Sample #5 grind; SEM.

The microstructures of slag samples and phase contrast brightness distribution histograms are presented in Figure 1, Figure 2, Figure 3, Figure 4 and Figure 5. An analysis of the morphology of inclusions and phase contrast allowed us to identify the quantitative composition of the slag. This technique is typical for the electron microscopy of minerals. The histograms presented in Figure 1c, Figure 2c, Figure 3c, Figure 4c, and Figure 5c were obtained as the averages of 10 view fields for each of five metallographic sections. The phase compositions of the slags were based on a histogram analysis and are presented in Table 2. One can see that these samples have similar sets of phases, but phase relationship varies in a wide range. 

To detect the amounts of vanadium with different oxidation degrees, we used XPS. It is well known that the kinetic energy of photoelectrons emitted from the inner shells of atoms depends on the chemical bonds or oxidation degree of the atom; the so-called chemical shift of the XPS characteristic line allowed us to identify the oxidation degree of the element in a compound. The number of registered photoelectrons (intensity of the peak) corresponding to a given element depends on the element’s concentration plus the yield cross-section σ of electrons located on the corresponding orbitals of the atom. Taking into account σ(O1s) = 2.93, σ(V2p_3/2_) = 6.37, and σ(V2p_1/2_) = 3.29, the oxygen line intensity should be 2.17 times lower than the V2p_3/2_ line and 1.12 times lower than the V2p_1/2_ line at equal concentrations of O and V. By using the correction for σ, one can quantitatively determine the element content from XPS spectra. For example, by taking the relationship of integral intensities of vanadium and oxygen lines (I_V_/I_O_) into account_,_ one can calculate their content relation in the sample. Based on XPS line fine structure analysis, we can get fractions of the element with various oxidation degrees. This technique is widely used in XPS as a standard one (Electron Spectroscopy for Chemical Analysis, ESCA) [30,31,32].

Photoelectron spectroscopy allowed us to determine the so-called “chemical shifts” of the characteristic elemental lines. These chemical shifts of the electron binding energies are stipulated by a change of the oxidation degrees of the elements or a change in the number of valence electrons transferred from donor atoms (V) to acceptor atoms (O) at the formation of the chemical compound.

**Figure 6:**
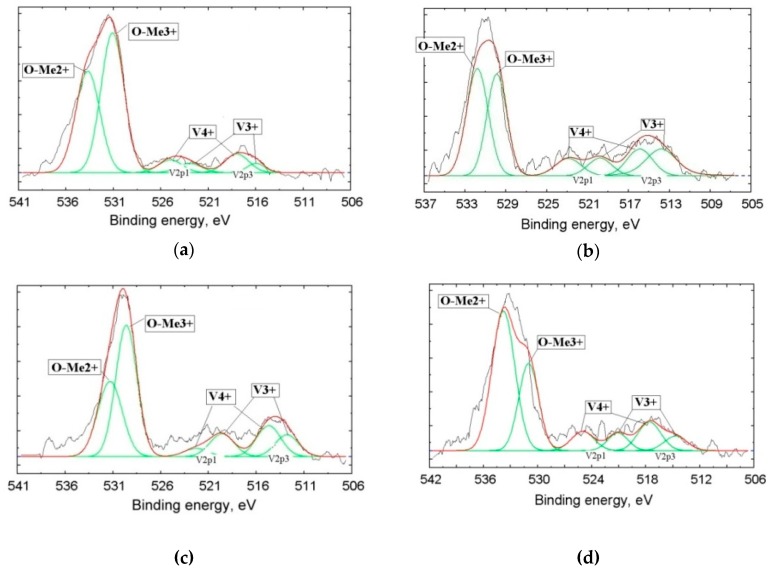
Fine structure of donor atoms (V) XPS (X-ray photoelectron spectroscopy) lines 2p_3/2–1/2_ and O1s obtained from Samples 2 (**a**), 3 (**b**), 4 (**c**), and 5 (**d**).

Figure 6 and Table 3 present the results of the vanadium slags studied by XPS. The figure presents the lines of the inner shells O1s with a binding energy in the vicinity of 530 eV and the lines of the V2p doublet with binding energies about 513 eV (V2p_3/2_) and 520 eV (V2p_1/2_). Each of the V2p doublet components corresponds to a definite oxidation degree of vanadium: The V^4+^ binding energies are E_b_ = 513.6 and 519.8 eV, while the components at E_b_ = 516.4 and 522.8 eV manifest in the presence of V^3+^. By measuring the integral intensities of components, we can determine a part of V in the definite oxidation state in the sample.

The presence of V^4+^ in large quantities in metallurgical slags was firstly established in this research. These data, approved by XPS, contradict common opinion regarding the singular presence of V^3+^ in the slag [11,12,13] 

The oxygen line O1s, which has distinctive asymmetry, demonstrates the overlapping of signals from the oxygen bound with metals with +3 and +2 oxidation degrees: Me^3+^ corresponds to E_b_ = 530.4 ± 0.2 eV, while Me^2+^ is associated with E_b_ = 531.8 ± 0.2 eV.

The oxidation degree data in the slags with a quantitative analysis of element contents from the XPS line fine structure analysis are presented in Table 3. These data qualitatively confirm the presence of a mixture of various elements oxides in probes with a preferential content of metals with a +2 oxidation degree.

## 4. Conclusions

The application of scanning electron microscopy, together with digital image analysis, allowed us to quickly estimate the relationships of the multiple phases presented in slag samples.

For the first time, the fact that V^4+^ is present in large quantities in metallurgical slags was approved by an XPS analysis. Such information is critically important for vanadium extraction technology improvements.

The integrated application of surface analysis methods such as scanning electron microscopy and X-ray photoelectron spectroscopy allows us to determine the content of vanadium in multiple oxidation degrees in slags and to properly select the chemicals and parameters of chemical processes for its fullest extraction.

## Figures and Tables

**Table 1:** The elemental composition of slags studied, wt. %.

Sample #	Al	Ca	Cr	Fe	Mg	Mn	P	S	Si	Ti	V	O
1	0.68	1.56	2.58	25.3	0.93	11.6	0.012	0.011	6.57	7.48	12.1	balance
2	0.88	2.84	2.87	23.2	0.98	11.6	0.021	0.013	6.11	5.96	13.6	balance
3	1.27	1.55	2.79	24.0	1.83	10.6	0.016	0.006	5.23	7.74	13.2	balance
4	1.86	1.20	2.37	29.1	2.48	8.6	0.040	0.005	4.69	6.75	11.6	balance
5	2.27	1.97	2.45	22.4	1.78	9.3	0.027	0.013	8.05	5.04	12.0	balance

**Table 2:** Phase composition of slag samples according to SEM data.

Sample #	Phase Fraction, %			
	FeO·Fe_2_O_3_	MnO·V_X_O_Y_	TiO_2_	Fe
1	balance	11.6 ± 1.1	9.2 ± 1.2	4.2 ± 0.7
2	balance	17.8 ± 1.6	8.2 ± 1.2	4.5 ± 0.8
3	balance	23.1 ± 1.8	1.7 ± 0.8	10.9 ± 1.4
4	balance	8.0 ± 1.0	21.6 ± 1.8	3.8 ± 0.7
5	balance	6.5 ± 0.9	24.1 ± 1.8	3.5 ± 0.6

**Table 3:** Content of elements with different oxidation degrees in slags.

Sample #	Binding Energy, eV	Oxidation Degree	Volume Fraction, %
2	515.3; 522.4	V^3+^	65.8
	517.6; 524.5	V^4+^	34.2
	531.1	Me^2+^	43.1
	533.7	Me^3+^	56.9
3	513.6; 519.8	V^3+^	56.4
	516.4; 522.8	V^4+^	43.6
	530.3	Me^2+^	42.0
	531.6	Me^3+^	58.0
4	512.8; 519.6	V^3+^	49.6
	514.7; 522.3	V^4+^	50.4
	529.6	Me^2+^	39.6
	531.3	Me^3+^	60.4
5	513.5; 520.0	V^3+^	34.8
	516.5; 523.1	V^4+^	65.2
	530.4	Me^2+^	34.1
	532.0	Me^3+^	65.9
